# Changes in synaptic inputs to dI3 INs and MNs after complete transection in adult mice

**DOI:** 10.3389/fncir.2023.1176310

**Published:** 2023-07-05

**Authors:** Sara Goltash, Shannon J. Stevens, Emine Topcu, Tuan V. Bui

**Affiliations:** Department of Biology, Brain and Mind Research Institute, University of Ottawa, Ottawa, ON, Canada

**Keywords:** spinal cord injury, dI3 interneurons, motoneurons, synaptic inputs, sensorimotor integration

## Abstract

**Introduction:**

Spinal cord injury (SCI) is a debilitating condition that disrupts the communication between the brain and the spinal cord. Several studies have sought to determine how to revive dormant spinal circuits caudal to the lesion to restore movements in paralyzed patients. So far, recovery levels in human patients have been modest at best. In contrast, animal models of SCI exhibit more recovery of lost function. Previous work from our lab has identified dI3 interneurons as a spinal neuron population central to the recovery of locomotor function in spinalized mice. We seek to determine the changes in the circuitry of dI3 interneurons and motoneurons following SCI in adult mice.

**Methods:**

After a complete transection of the spinal cord at T9-T11 level in transgenic Isl1:YFP mice and subsequent treadmill training at various time points of recovery following surgery, we examined changes in three key circuits involving dI3 interneurons and motoneurons: (1) Sensory inputs from proprioceptive and cutaneous afferents, (2) Presynaptic inhibition of sensory inputs, and (3) Central excitatory glutamatergic synapses from spinal neurons onto dI3 INs and motoneurons. Furthermore, we examined the possible role of treadmill training on changes in synaptic connectivity to dI3 interneurons and motoneurons.

**Results:**

Our data suggests that VGLUT1^+^ inputs to dI3 interneurons decrease transiently or only at later stages after injury, whereas levels of VGLUT1^+^ remain the same for motoneurons after injury. Levels of VGLUT2^+^ inputs to dI3 INs and MNs may show transient increases but fall below levels seen in sham-operated mice after a period of time. Levels of presynaptic inhibition to VGLUT1^+^ inputs to dI3 INs and MNs can rise shortly after SCI, but those increases do not persist. However, levels of presynaptic inhibition to VGLUT1^+^ inputs never fell below levels observed in sham-operated mice. For some synaptic inputs studied, levels were higher in spinal cord-injured animals that received treadmill training, but these increases were observed only at some time points.

**Discussion:**

These results suggest remodeling of spinal circuits involving spinal interneurons that have previously been implicated in the recovery of locomotor function after spinal cord injury in mice.

## Introduction

Spinal cord injury (SCI) is a debilitating condition where the communication between supraspinal regions and the spinal cord is severed, causing significant motor, sensory and autonomic function disruptions. Locomotor function is often impaired or lost following SCI due to the loss of motor commands conveyed by descending inputs. However, experiments in vertebrates have repeatedly shown that despite the loss of descending locomotor commands, the spinal cord contains the neural machinery that is capable of generating some level of locomotor activity through various means of stimulation, whether electrical, chemical, or sensory (Lavrov et al., 2008; Cowley et al., 2015; Gerasimenko et al., 2019, 2002; Zhang et al., 2021; Kathe et al., 2022). For instance, studies in cats and rodents have shown that after a complete SCI, a treadmill training regimen can provide rhythmic sensory input to the spinal circuits that lead to the reactivation of these circuits and patterns of muscle activation that produce stepping (Tillakaratne et al., 2000; Courtine et al., 2009; Khristy et al., 2009; Roy et al., 2012; Shah et al., 2013; Takeoka et al., 2014; Takeoka and Arber, 2019).

In several cases, the recuperation of locomotor activity after SCI is associated with the plasticity of the spinal cord (Frigon and Rossignol, 2006; Rossignol, 2006; Rossignol and Frigon, 2011). This plasticity likely facilitates the activation of spinal circuits for locomotion in the injured spinal cord. Propriospinal neurons have been suggested as sites of plasticity within the spinal cord (Laliberte et al., 2019). Propriospinal neurons play a role in locomotion by receiving inputs from descending locomotor pathways and propagating motor commands rostrocaudally to locomotor circuits via short or long, ipsilateral, or commissural axons. They have also been implicated in the recovery of locomotion after injury. Following a hemisection or a staggered lesion, propriospinal neurons have been shown to form detour circuits around the lesion and form new connections between the descending corticospinal or reticulospinal tracts and propriospinal neurons in distal regions (Bareyre et al., 2004; Bareyre, 2008; Courtine et al., 2008; Martinez and Rossignol, 2013; May et al., 2017; Asboth et al., 2018). These detour circuits enable the rerouting of motor commands to spinal circuits distal to the injury in incomplete spinal cord injuries.

However, the formation of detours around spinal lesions is much less likely after complete SCI, where regeneration of axons across the lesion sites remains a significant challenge (Cummings et al., 1981; Tillakaratne et al., 2010). Despite the lack of regenerated axons across the site of injury, animal models of complete SCI can also exhibit some recovery of locomotor function. Again, plasticity in spinal circuits below the lesion has been implicated in promoting locomotor activity in the absence of supraspinal commands (Edgerton et al., 2004; Bareyre, 2008; Flynn et al., 2011; Rossignol and Frigon, 2011; Côté et al., 2012; Gerasimenko et al., 2019; Swieck et al., 2019).

Plasticity of spinal circuits that improves or increases sensory feedback to spared spinal locomotor circuits distal to the level of injury may be particularly beneficial to promoting locomotor recovery, especially after complete spinal transections. Loss of descending inputs leaves sensory pathways as one of the few remaining entry points to spinal locomotor circuits (Bouyer and Rossignol, 2003; Lavrov et al., 2008; Sławińska et al., 2012; Takeoka et al., 2014). For example, during locomotor training on a treadmill, the continual mechanical movement of the paws of animals may enhance sensory feedback from the cutaneous and proprioceptive sensory neurons onto the spinal locomotor networks. Elimination of sensory inputs has been shown to impair gait control and locomotor recovery during treadmill walking in cats and rodents (Bouyer and Rossignol, 2003; Edgerton et al., 2007; Frigon and Rossignol, 2008; Sławińska et al., 2012; Takeoka et al., 2014; Takeoka and Arber, 2019).

Quantification of VGLUT1^+^ synapses, which include proprioceptive and low-threshold sensory afferents as well as corticospinal tract boutons in the spinal cord (Alvarez et al., 2004), suggests changes in the density of sensory afferents to some but not all MNs after complete lumbar spinal transection in rats (Khalki et al., 2018). The same study observed changes in the percentage of these VGLUT1^+^ boutons contacted by axoaxonic GAD65-stained boutons. Training after SCI was also observed to change presynaptic and postsynaptic inhibition levels in the same cohort of animals. Reductions in GABAergic boutons contacting VGLUT1^+^ boutons were also reported after complete sacral transections (Kapitza et al., 2012). These prior studies focused mainly on spinal motoneurons, which are the direct interface between the CNS and the skeletomuscular system. However, many populations of spinal neurons also receive sensory inputs (Brownstone and Bui, 2010), and some of these populations have been implicated in the recovery of locomotor function (Bui et al., 2016; Kathe et al., 2022). Whether there is plasticity in the integration of this sensory feedback by these and other populations of spinal neurons that are crucial to the recovery of locomotor function remains to be determined.

We have previously identified a population of dorsal interneurons, called dI3 interneurons (dI3 INs), as an integral part of the recovery of locomotor function in spinalized mice. dI3 INs are a population of spinal neurons residing in laminae V–VII of the spinal cord (Bui et al., 2013). They are characterized by the expression of Isl1 (Bui et al., 2016; Capelli et al., 2017), a LIM-homeodomain transcription factor that is also expressed in various tissues and populations of neurons, including motoneurons and sensory neurons (Pfaff et al., 1996; Liem et al., 1997) but is exclusively expressed by dI3 INs in the intermediate spinal cord.

dI3 INs form sensorimotor circuits linking sensory afferents and neurons for motor control in the spinal cord (Bui et al., 2013, 2016; Laliberte et al., 2022). Immunohistochemical labeling of VGLUT1^+^ has shown that dI3 INs are contacted by primary sensory afferents (Alvarez et al., 2004), and electrophysiological experiments have confirmed this observation as dI3 INs received monosynaptic input from proprioceptive and low threshold cutaneous afferents (Bui et al., 2013), many of which are under the control of axoaxonic GABAergic inputs (Laliberte et al., 2022). Furthermore, dI3 INs are glutamatergic and project ipsilaterally within the spinal cord targeting motoneurons and spinal locomotor circuits (Bui et al., 2016).

The integration of proprioceptive and mechanoreceptive feedback for motor control suggests that dI3 INs could be involved in promoting locomotor function after SCI. Indeed, when we silenced dI3 INs glutamate transmission using transgenic approaches, spinalized mice showed attenuated locomotor function on the treadmill (Bui et al., 2016). Given prior evidence suggesting possible plasticity of spinal circuitry following SCI, we asked whether the circuitry of dI3 INs could also be altered. We thus investigated changes in synaptic inputs related to sensory integration by dI3 INs and MNs following a complete transection of the spinal cord in adult mice. Our results suggest that some changes in synaptic connectivity to dI3 INs and MNs occur, though the relationship between those changes and recovery levels is currently difficult to ascertain.

## Materials and methods

### Animals

All animal procedures were approved by the University of Ottawa Animal Care Committee and conform to the guidelines put forth by the Canadian Council for Animal Care. Animals used in experiments ranged in age from adult 3 to 6 months old. Both male and female mice were used in this study. We used Isl1-Cre; Rosa26-YFP transgenic line of mice, herein referred to as Isl1:YFP mice, which allowed us to visualize dI3 INs through the expression of yellow fluorescent protein (YFP).

### Surgical details of spinal cord injury

Complete transections were performed at T9-T10 under isoflurane anesthesia. This injury interrupts all descending supraspinal pathways. Sterile surgical foam (Surgifoam, Johnson Johnson Medtech) was placed in the spinal space to prevent any re-growth of the axons across the lesion. Animals were individually housed, given analgesic (Buprenorphine, Ceva Animal Health) for 3 days, and allowed to recover for at least 1 week prior to treadmill training. Animals were monitored thrice daily, and bladders were expressed manually thrice daily. Humane endpoints were defined as self-mutilation, improper feeding, decreased grooming, ataxia, or loss of body weight >20%.

Locomotor training on a custom-made treadmill was provided twice a week, with an additional testing session per week. Animals were allowed to habituate to the treadmill environment before training and then trained on the treadmill (belt speed of 10 cm/s) with minimal weight support to ensure continual contact with the treadmill belt. On the testing day, mice walking on the treadmill were filmed from the side using a Basler ace acA640—750 μm camera to record at 60 frames per second for 3 min. Views of the mice from the side and the bottom were captured as the treadmill was equipped with a transparent belt and a mirror below. In addition to the session walking with no weight support, a subset of mice also had a session walking in a more upright position by providing manual body weight support during the weekly testing. We previously observed that recovery of rhythmic hindlimb movements plateaus after about 2 months (Bui et al., 2016). Based upon these data, we collected the lumbar spinal cord in mice after 6 days post-injury (dpi), 2–3 weeks post-injury (wpi), 4–6 and 10–12 wpi of locomotor training. SCI mice were sacrificed at these time points, and spinal cords were removed for immunohistochemistry.

### Perfusion and immunohistochemistry

Mice were injected with 120 mg/kg pentobarbital sodium and, after sedation, checked for the presence of a toe pinch reflex. Following complete anesthesia, animals were cut open and transcardially perfused with PBS for 5 min and then with ice-cold 4% paraformaldehyde for another 5 min or until the liver was clear of blood. Next, the spinal cord was removed and post-fixed in fresh 4% PFA overnight. The next day, the spinal cord was stored in 30% sucrose for cryoprotection and, after a few days, embedded in OCT cryostat sectioning medium and stored at −80°C. When ready for sectioning, the spinal cord was cut in transverse sections on a cryostat (40–50 μm) and collected as free-floating sections. Sections were washed three times with PBS, blocked for 1 h in PBS with 0.3% triton-X100 (PBST) and 5% normal goat serum, and then incubated overnight at 4°C with primary antibodies in PBST plus 5% serum. The following day, sections were washed three times with PBS, incubated with Alexa Fluor-conjugated secondary antibodies for 3 h at room temperature and washed three final times in PBS. Antibodies used for fluorescent immunohistochemistry were as follows: Chicken anti GFP: (Abcam Cat# ab13970, RRID:AB_300798), Guineapig anti VGLUT1: (Millipore Cat# AB5905, RRID:AB_2301751), Guineapig anti VGLUT2: (Millipore Cat# AB5907, RRID:AB_2301731) and mouse anti-GAD65/67 (3 μg/ml; Developmental Studies Hybridoma Bank; RRID: AB 2314499). Sections were mounted onto Superfrost (Fisher Scientific) slides with Immu-Mount (Fisher Scientific), and a coverslip was applied.

### Microscopy and synaptic quantification

Olympus FV1000 BX61 confocal laser scanning microscope (RRID:SCR_020337) was used to acquire the images and optical sections were collected in z-stacks. Fluoview software (Olympus) was used to analyze the images. From each mouse, 20 dI3 INs and 20 MNs were selected, and the number of boutons was quantified manually using orthogonal views to confirm apposition onto the cell body. For quantification of sensory synapses on dI3 INs and MNs, VGLUT1^+^ boutons were quantified on the entire soma and proximal dendrites (up to 100 μm from the soma) on confocal images. Similarly, VGLUT2^+^ boutons were counted to estimate the number of central excitatory synapses onto dI3 INs and MNs. In addition, GAD65^+^ boutons contacting VGLUT1^+^ terminals were counted, and the fraction of VGLUT1^+^ boutons with GAD65^+^ boutons was calculated to estimate presynaptic inhibition of sensory inputs to dI3 INs and MNs. To estimate the surface area of the soma, thresholding of the image was applied using ImageJ software. Specifically, a rectangle was drawn around the cell of interest and the threshold was adjusted until the surface of the cell had become white while the background remained black. The area of the soma was then measured. To measure the size of the boutons, the optical section containing the largest area of each button was selected and the maximum diameter of the bouton was measured using ImageJ.

### Automated detection of stepping function

Videos of stepping function during weekly testing sessions were analyzed using DeepLabCut (Version: 2.2.0.6, RRID:SCR_021391) (Mathis et al., 2018). The training of the network involved using the graphical interface provided by DLC to label various body parts. Several body parts from the side and bottom view were labeled, though for the purpose of this study, only the following body parts were analyzed: nose, ear, left knee, left hind toes, left ankle, and tail base. For the training of the network, one video from each mouse filmed at the 2–3, 4–6, and 10–12 wpi time points were chosen per time point. Around 20–40 frames were labeled per video, and the label diameter was set to 5 pixels. Training the network was done with Google Colab using the ResNet-50 network with the number of iterations set to 200,000 and an accepted range of error set to 3 pixels.

The trained network was then used to analyze all the testing videos to estimate the horizontal and vertical coordinates of each body part detected. The validity of the calculated coordinate was based on a likelihood probability with a threshold of 0.5 for SCI mice and 0.8 for sham mice. For sham mice, video frames where mice were sitting or standing up were excluded if they did not meet the following criteria: (1) The difference between the vertical coordinates of the nose and ear to the tails was between the median value ± two standard deviations for the video analyzed. (2) The horizontal coordinates of both the nose and ear are more forward than the tail base.

The left ankle angle was calculated as the angle between the left knee, left ankle, and left hind toe. We used the left ankle angle to estimate the number of steps. The ankle angle for SCI mice on a treadmill without weight was frequently extended between 90 and 180°. Stepping motions of their limbs flexed the ankle resulting in a rapid decrease of the ankle angle below 90°. Thus, we detected minimum peak angles using a 30-frame-long moving time window. The occurrences of local minima below 90° were identified as steps, and the number of steps per minute across test sessions was estimated per mouse analyzed. For sham mice, local minima and maxima of ankle angles were determined using a 10-frame-long moving time window. Due to the occasional presence of consecutive minima without an interposed maximum or vice-versa, the number of steps was calculated as the arithmetic mean of the number of local minima and maxima in each video.

### Statistical analysis

GraphPad Prism 9.2.0 (RRID:SCR_002798) was used to perform all statistical analyses. Data are reported as mean ± SD. For all tests, the level of statistical significance was set at *p* < 0.05 and indicated by asterisks in the figures. Comparisons involving all means within a group were made using one-way ANOVA followed by comparisons between pairs of groups using Tukey’s multiple comparison test. Comparisons involving only a subset of all possible pairs (trained vs. untrained at each time point) within a group were performed using *t*-tests with Bonferroni’s multiple-comparisons correction. Correlations between synaptic inputs and stepping function were analyzed using the non-parametric Spearman Correlation test.

## Results

Thirty-eight transgenic Isl1:YFP mice, 21 in the trained group (Sex: 8 M, 13 F, Average weight = 22.09 ± 2.37 g) and 17 in the untrained group (Sex: 9 M, 8 F, Average weight = 21.5 ± 2.4 g) underwent a complete transection of the spinal cord at T9-T10. The transection occurred at age 3.62 ± 0.76 months. Data from 3 mice were not included due to incomplete lesions of the spinal cord or walking performance on the treadmill that was very close to sham levels shortly after the injury.

### Changes in sensory inputs to dI3 INs and MNs

**FIGURE 1:**
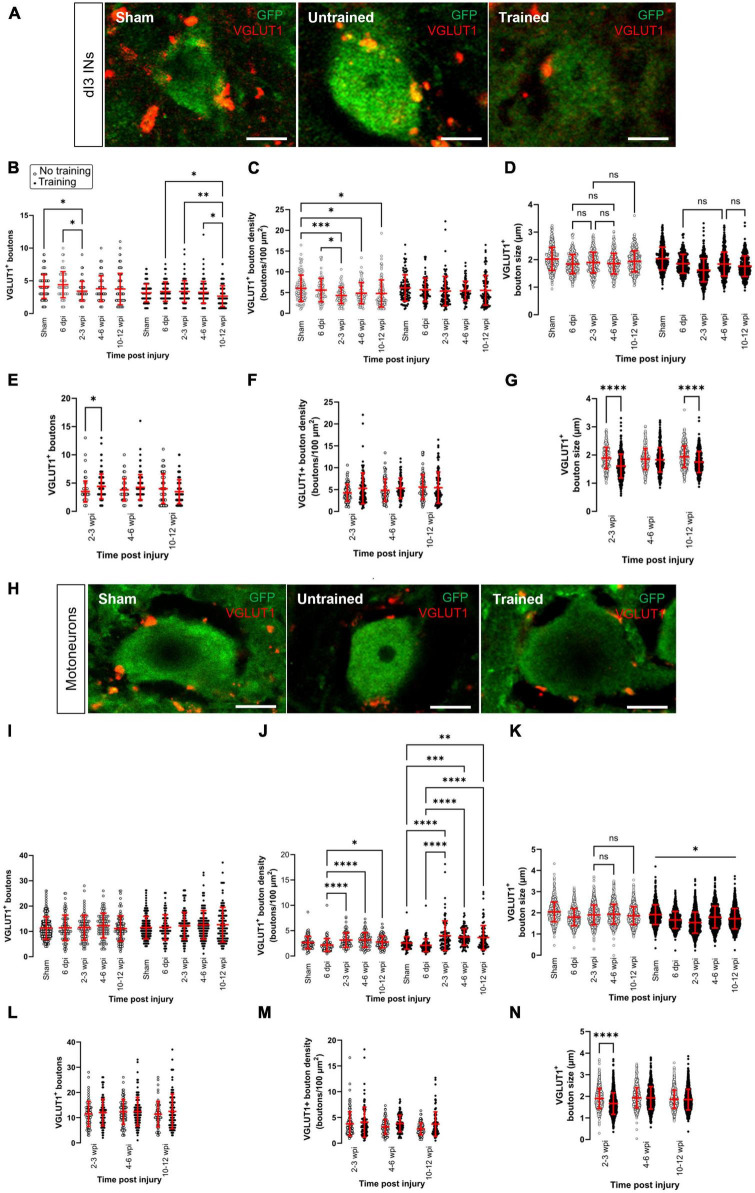
Changes in VGLUT1^+^ sensory inputs to dI3 INs and MNs following SCI. Immunostaining of sensory boutons from proprioceptive and mechanoreceptive afferents onto **(A)** dI3 INs and **(H)** MNs from 10 to 12 wpi animals (except for sham). Number of VGLUT1^+^ boutons per lumbar **(B)** dI3 IN and **(I)** MNs; VGLUT1^+^ bouton density per lumbar **(C)** dI3 IN and **(J)** MNs; and size of VGLUT1^+^ boutons to **(D)** dI3 INs and **(K)** MNs in mice that did not receive (left) and received treadmill training (right). The data in left and right panels of **(B–D,I–K)** for sham and 6 dpi are the same as neither group underwent training post-surgery. Comparing the effect of locomotor training on the number of VGLUT1^+^ boutons, their density, and their size, respectively, for **(E–G)** dI3 INs and **(L–N)** MNs at different time points post-injury. Circles denote counts for individual cells. Red lines denote ± SD. *p ≤ 0.05, ^**^p ≤ 0.01, ^***^p ≤ 0.001, ^****^p ≤ 0.0001 [Tukey’s multiple comparison test following one-factor ANOVA for **(B–D,I–K)**; *t*-tests with Bonferroni’s multiple-comparisons correction for **(E–G,L–N)**]. In all of **(D)** and just **(K)** left, all pairwise comparisons are significant unless noted by ns. In **(K)** right, all pairwise comparisons are statistically significant. Scale bar in images = 10 μm.

We first sought to determine changes in sensory inputs to dI3 INs and MNs. dI3 INs and MNs were identified by yellow fluorescent protein (YFP) expression in Isl1:YFP transgenic mice. To characterize sensory terminals onto dI3 INs and MNs, we used an antibody against VGLUT1^+^ (Figures 1A, H) a vesicular glutamate transporter selectively expressed in central boutons of proprioceptors and low-threshold mechanoreceptors (Alvarez et al., 2004). VGLUT1^+^ sensory boutons on the surface of dI3 INs and MNs were quantified at different time points post-injury in two groups of mice with and without treadmill training.

The number of VGLUT1^+^ boutons contacting dI3 INs changed after SCI in the untrained and trained animals (Figure 1B; *N* = 17 untrained animals and *N* = 21 trained animals; one-factor ANOVA, *p* = 0.0089 for untrained animals, *p* = 0.0079 for trained animals). In the untrained group, differences were limited to the 2–3 wpi time point, which was significantly decreased compared to sham and the 6 dpi time point (Figure 1B left panel; *N* = 10 sham animals, *N* = 4 at 6 dpi and 2–3 wpi, Tukey’s multiple comparison test: *p* = 0.0416 and *p* = 0.0124, respectively). In dI3 INs from trained animals, the number of VGLUT1^+^ boutons was significantly decreased at 10–12 wpi compared to 6 dpi, 2–3 wpi and 4–6 wpi (Figure 1B right panel; *N* = 4 at 6 dpi, *N* = 6 at 2–3 and 4–6 wpi, *N* = 5 at 10–12 wpi, Tukey’s multiple comparison test: *p* = 0.0245, *p* = 0.008, *p* = 0.01498, respectively). Pairwise comparisons between the untrained and trained groups suggest a greater number of VGLUT1^+^ boutons at 2–3 wpi in the trained mice compared to untrained mice (Figure 1E; *N* = 4 untrained animals, *N* = 6 trained animals, *t*-tests with Bonferroni’s multiple-comparisons correction: *p* = 0.0007).

We then normalized the number of VGLUT1^+^ boutons by an estimate of soma area and found changes only in untrained animals (Figure 1C; *N* = 17 untrained animals and *N* = 18 trained animals; one-factor ANOVA, *p* = 0.003 for untrained animals, *p* = 0.4525 for trained animals). In the untrained group, bouton density was decreased between sham and the longer-term injury time points 2–3, 4–6, and 10–12 wpi (Figure 1C left panel; *N* = 5 sham and at 10–12 wpi, *N* = 4 at 2–3 and 4–6 wpi, Tukey’s multiple comparison test: *p* = 0.0004, *p* = 0.0421, and *p* = 0.0160, respectively). Pairwise comparisons of VGLUT1^+^ bouton density on dI3 INs from trained vs. untrained mice failed to show any differences (Figure 1F; *N* = 4–5 untrained and trained animals, *t*-tests with Bonferroni’s multiple-comparisons correction).

We also measured the size of VGLUT1^+^ boutons to dI3 INs at different time points after injury. We found very small but statistically significant differences in the size of boutons in the untrained and trained animals (Figure 1D; *n* = 273–388 boutons in untrained animals and *n* = 346–459 boutons in trained animals; one-factor ANOVA, *p* < 0.0001 for untrained and trained animals). The VGLUT1^+^ boutons to dI3 INs in sham (mean = 2.0 ± 0.4 μm) was similar or larger than VGLUT1^+^ boutons to dI3 INs in SCI mice, which ranged in average from 1.6 to 1.9 μm. Pairwise comparisons of VGLUT1^+^ bouton size on dI3 INs from trained vs. untrained mice suggest differences at 2–3 dpi (Figure 1G; *n* = 273 synapses in untrained animals and 459 synapses in trained animals, *t*-tests with Bonferroni’s multiple-comparisons correction, *p* < 0.0001) and at 10–12 wpi (Figure 1G; *n* = 375 synapses in untrained animals and 346 synapses in trained animals, *t*-tests with Bonferroni’s multiple-comparisons correction, *p* < 0.0001).

No changes were observed in the number of VGLUT1^+^ boutons on motoneurons in either of the trained and untrained animals (Figure 1I; *N* = 17 untrained animals and *N* = 21 trained animals; one-factor ANOVA, *p* = 0.54 for untrained animals, *p* = 0.26 for trained animals). Pairwise comparisons between the untrained and trained groups at different time points failed to reveal any differences in VGLUT1^+^ boutons to motoneurons due to training (Figure 1L, *t*-tests with Bonferroni’s multiple-comparisons correction). On the other hand, differences were revealed when analyzing the density of VGLUT1^+^ bouton on motoneurons. VGLUT1^+^ bouton density on motoneurons was higher at the longer-term injury time points 2–3, 4–6, and 10–12 wpi than 6 dpi in untrained animals (Figure 1J left panel; *N* = 4 at 6 dpi, 2–3 wpi, and 4–6 wpi, *N* = 5 dpi at 10–12 wpi, Tukey’s multiple comparison test: *p* < 0.0001, *p* < 0.0001, and *p* = 0.0208, respectively). Similarly, VGLUT1^+^ bouton density on motoneurons was higher at the longer-term injury time points 2–3, 4–6, and 10–12 wpi than both sham and 6 dpi in trained animals (Figure 1J right panel; *N* = 4 at 6 dpi, 2–3 wpi, and 4–6 wpi, *N* = 5 dpi sham and at 10–12 wpi, Tukey’s multiple comparison test: *p* < 0.0001, *p*−0.0004, and *p* = 0.0021, respectively in comparison to sham, *p* < 0.0001 for all comparisons between 2–3, 4–6, and 10–12 wpi and 6 dpi). Pairwise comparisons of VGLUT1^+^ bouton density on MNs from trained vs. untrained mice failed to show any differences (Figure 1M; *t*-tests with Bonferroni’s multiple-comparisons correction).

An analysis of the size of VGLUT1^+^ boutons to MNs at different time points after injury revealed similar findings to dI3 INs. We found very small but statistically significant differences in the size of boutons in the untrained and trained animals (Figure 1K; *n* = 912–1110 boutons in untrained animals and *n* = 912–1248 boutons in trained animals; one-factor ANOVA, *p* < 0.0001 for untrained and trained animals). The VGLUT1^+^ boutons to MNs in sham (mean = 2.0 ± 0.5 μm) was larger than VGLUT1^+^ boutons to MNs in SCI mice, where the average VGLUT1^+^ boutons ranged from 1.6 to 1.9 μm. Pairwise comparisons of VGLUT1^+^ bouton size on MNs from trained vs. untrained mice revealed a difference at 2–3 wpi (Figure 1N; *n* = 916 synapses in untrained animals and 1216 synapses in trained animals, *t*-tests with Bonferroni’s multiple-comparisons correction, *p* < 0.0001).

Overall, our data suggest that changes in the number of sensory inputs were limited to dI3 INs and not motoneurons. The changes in the number of VGLUT1^+^ boutons to dI3 INs were only observed at specific time points; the timing depended on whether the animal received treadmill training. A difference in the number of VGLUT1^+^ inputs between trained and untrained animals was only seen in dI3 INs and only at 2–3 wpi. When accounting for soma size, MNs showed an increase in VGLUT1^+^ bouton density at longer time points after SCI, whereas untrained dI3 INs showed a decrease in VGLUT1^+^ bouton density at longer time points after SCI. An analysis of bouton size suggests that VGLUT1^+^ boutons to dI3 INs and MNs are smaller at many time points after injury compared to sham.

### Changes in central excitatory inputs

**FIGURE 2:**
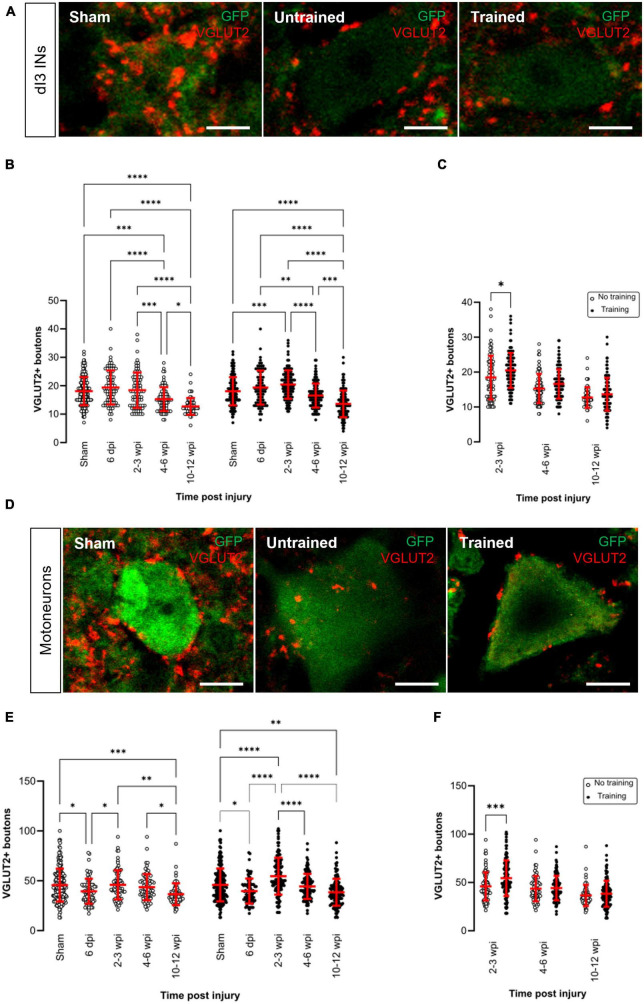
Changes in VGLUT2^+^ central excitatory inputs to dI3 INs and MNs following SCI. Immunostaining of excitatory boutons from spinal neurons onto **(A)** dI3 INs and **(D)** MNs from 10 to 12 wpi animals (except for sham). **(B)** Number of VGLUT2 + boutons per lumbar dI3 IN and **(E)** MNs in the mice that did not receive (left) and received treadmill training (right). The data in left and right halves of **(B,E)** for sham and 6 dpi are the same as neither group underwent training post-surgery. **(C)** Comparing the effect of locomotor training in dI3 INs and **(F)** in MNs at different time points post-injury. Circles denote counts for individual cells. Red lines denote ± SD. *p ≤ 0.05, ^**^p ≤ 0.01, ^***^p ≤ 0.001; ^****^p ≤ 0.0001 [Tukey’s multiple comparison test following one-factor ANOVA for **(B,E)**; *t*-tests with Bonferroni’s multiple-comparisons correction for **(C,F)**]. Scale bar in images = 10 μm.

Next, we investigated whether there were changes in central excitatory inputs to dI3 INs and MNs after SCI. While sensory boutons in the spinal cord are labeled by VGLUT1^+^, excitatory glutamatergic boutons from central neurons are labeled exclusively by VGLUT2^+^ (Figures 2A, D) except for corticospinal inputs that are labeled by VGLUT1^+^ (Persson et al., 2006). We observed several changes in the dI3 INs (Figure 2B; *N* = 15 untrained animals and *N* = 21 trained animals; one-factor ANOVA, *p* < 0.0001 for untrained animals, *p* < 0.0001 for trained animals) and MNs of both untrained and trained animals (Figure 2E; *N* = 15 untrained animals and *N* = 21 trained animals; one-factor ANOVA, *p* < 0.0001 for untrained animals, *p* < 0.0001 for trained animals).

In the untrained animals, there were no changes in VGLUT2^+^ inputs to dI3 INs until 4–6 wpi where there was a significant decrease compared to sham, 6 dpi and 2–3 wpi (Figure 2B left panel; *N* = 10 sham and *N* = 4 at 6 dpi, 2–3 wpi and 4–6 wpi, Tukey’s multiple comparison test: *p* = 0.0003, *p* < 0.0001, *p* = 0.0009, respectively) and a further decrease at 10–12 wpi (Figure 2B left panel; *N* = 3, Tukey’s multiple comparison test: *p* = 0.026).

On the other hand, in the trained animals, there was an initial increase in VGLUT2^+^ inputs to dI3 INs at 2–3 wpi compared to sham (Figure 2B right panel; *N* = 10 sham and *N* = 6 animals at 2–3 wpi, Tukey’s multiple comparison test: *p* = 0.0005) but this increased excitatory inputs did not last long and was decreased at 4–6 wpi (Figure 2B right panel; *N* = 6 animals, *p* < 0.0001) and further decreased at 10–12 wpi (Figure 2B right panel; *N* = 5 animals, *p* < 0.0001). A pairwise comparison between the untrained and trained groups showed that there was a significant increase in the level of VGLUT2^+^ boutons to dI3 INs in the mice that received treadmill training at 2–3 wpi (Figure 2C; *N* = 4 untrained animals, *N* = 6 trained animals, *t*-tests with Bonferroni’s multiple-comparisons correction: *p* = 0.0119).

In the motoneurons of both untrained and trained mice, we observed an initial decrease in the level of VGLUT2^+^ boutons at 6 dpi compared to sham (Figure 2E; *N* = 10 sham and *N* = 4 animals at 6 dpi, Tukey’s multiple comparison test: *p* = 0.0115 untrained and *p* = 0.0193 trained) which later increased in both groups at 2–3 wpi (Figure 2E; *N* = 4 untrained and *N* = 6 trained animals, Tukey’s multiple comparison test: *p* = 0.0393 untrained and *p* < 0.0001 trained) but then decreased at 10–12 wpi (Figure 2E; *N* = 3 untrained and *N* = 5 trained animals, Tukey’s multiple comparison test: *p* = 0.0016 untrained and *p* < 0.0001 trained). A pairwise comparison between the untrained and trained groups showed that there was a significant increase in the level of VGLUT2^+^ boutons to MNs at 2–3 wpi in the mice that received treadmill training (Figure 2F; *N* = 4 untrained and *N* = 6 trained animals, *t*-tests with Bonferroni’s multiple-comparisons correction: *p* = 0.0007).

Overall, VGLUT2^+^ inputs to dI3 INs and MNs showed either an increase or decrease shortly after the injury, but at 10–12 wpi, VGLUT2^+^ levels to both types of neurons were lower when compared to sham animals in both trained and untrained animals.

### Changes in presynaptic inhibition of VGLUT1 + inputs

Excitatory inputs are important in reactivating the dormant circuits after SCI. However, inhibitory inputs gating the sensory afferents may also play a critical role in keeping a proper balance between excitation and inhibition within the spinal cord (Bertels et al., 2022). A prominent circuit that can gate the excitation of dI3 INs and MNs is formed by the spinal GABAergic neurons (referred to herein as GABApre neurons) that mediate presynaptic inhibition of VGLUT1-expressing central proprioceptive sensory terminals and cutaneous sensory afferents (Hughes et al., 2005; Betley et al., 2009). Therefore, we next looked at changes in presynaptic inhibition of sensory afferents onto dI3 INs and MNs after SCI.

**FIGURE 3:**
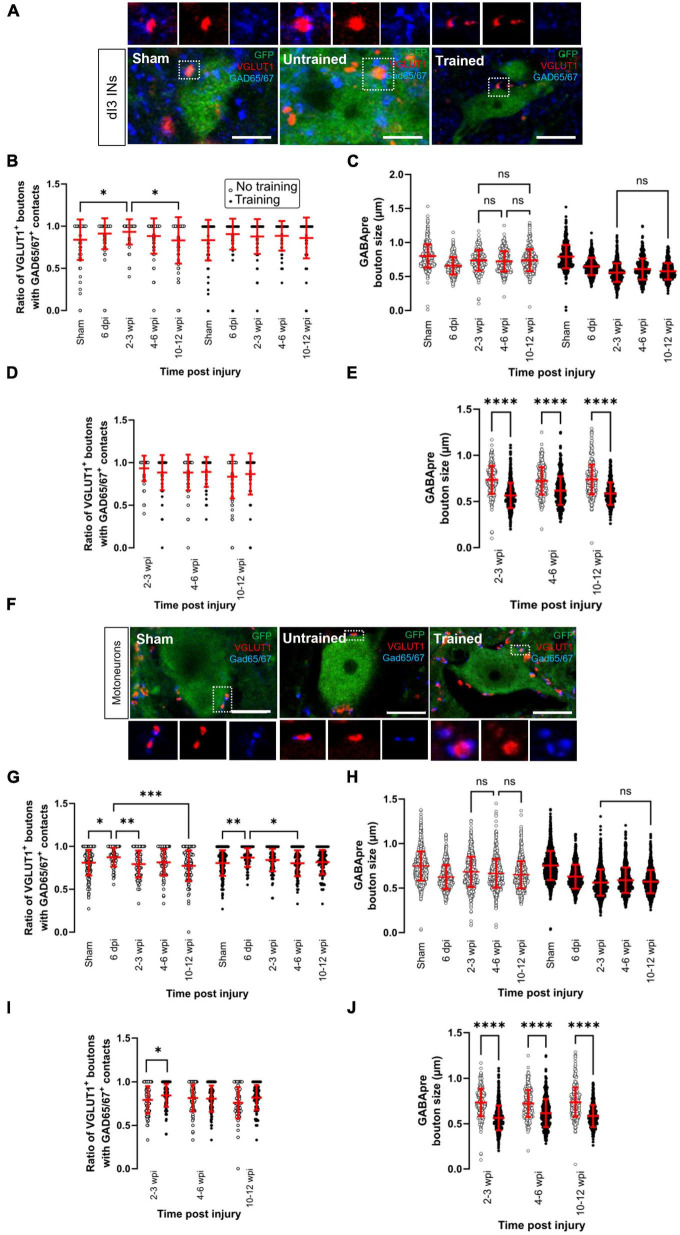
Changes in presynaptic inhibition of VGLUT1 + boutons onto dI3 INs and MNs following SCI. Presynaptic inhibition of sensory boutons is stained by GAD65/67^+^ and found on VGLUT1 + terminals to **(A)** dI3 INs and **(F)** MNs from 10 to 12 wpi animals (except for sham). Percent of VGLUT1 + inputs with GAD65^+^ contacts per lumbar **(B)** dI3 IN and **(G)** MN, and size of GAD65/67^+^ contacts on VGLUT1^+^ inputs to **(C)** dI3 INs and **(H)** MNs in the mice that did not receive (left panel) and received treadmill training (right panel). The data in left and right halves of **(B,C,G,H)** for sham and 6 dpi are the same as neither group underwent training post-surgery. Comparing the effect of locomotor training on the percentage of VGLUT1^+^ inputs with GAD65/67^+^ contacts and the size of GAD65/67^+^ contacts on VGLUT1^+^ inputs, respectively, to **(D,E)** dI3 INs and **(I,J)** MNs at different time points post-injury. Circles denote counts for individual cells. Red lines denote ± SD. *p ≤ 0.05, ^**^p ≤ 0.01, ^***^p ≤ 0.001, ^****^p ≤ 0.0001 [Tukey’s multiple comparison test following one-factor ANOVA for **(B,C,G,H)**; *t*-tests with Bonferroni’s multiple-comparisons correction for **(D,E,I,J)**]. **(C,H)** All pairwise comparisons are significant unless noted by ns. Scale bar in images = 10 μm.

GABApre terminals are distinguished from postsynaptic GABAergic terminals in the spinal cord by the dual expression of the two GAD isoforms, GAD65 and GAD67 (Erlander and Tobin, 1991). Thus, we used antibodies against GAD65/67 as molecular markers for terminals mediating presynaptic inhibition in the spinal cord and sought GAD65/67^+^ terminals in contact with VGLUT1^+^ sensory boutons (Figures 3A, F). In contrast, postsynaptic GABAergic inhibitory neurons directly target the cell body or dendrites of spinal neurons and are identified by GAD67 staining. Therefore, we quantified the percentage of VGLUT1^+^ boutons with GAD65/67^+^ boutons.

In the dI3 INs, changes in presynaptic inhibition were only observed in the untrained animals (Figure 3B; *N* = 17 untrained animals and *N* = 21 trained animals; one-factor ANOVA, *p* = 0.0035 for untrained animals, *p* = 0.077 for trained animals). There was a significant increase in the level of presynaptic inhibition of VGLUT1^+^ sensory inputs to dI3 INs from untrained animals at 2–3 wpi compared to sham (Figure 3B left panel; *N* = 10 in sham and *N* = 4 at 2–3 wpi, Tukey’s multiple comparison test: *p* = 0.0153) which later was reduced at 10–12 wpi to levels similar to sham levels (Figure 3B left panel; *N* = 5 at 10–12 wpi, Tukey’s multiple comparison test: *p* = 0.0225 for test between 2 and 3 wpi and 10–12 wpi; *p* = 0.9982 for test between sham and 10–12 wpi). A pairwise comparison between the trained and untrained animals at different time points failed to reveal any differences between the groups (Figure 3D; *t*-tests with Bonferroni’s multiple-comparisons correction).

On the other hand, we observed several changes in the MNs of both untrained and trained animals (Figure 3G; *N* = 17 untrained animals and *N* = 21 trained animals; one-factor ANOVA, *p* = 0.0005 for untrained animals, *p* = 0.0032 for trained animals). In the untrained group, there was an initial increase in the level of presynaptic inhibition at 6 dpi compared to sham (Figure 3G left panel, *N* = 10 sham, *N* = 4 animals at 6 dpi, Tukey’s multiple comparison test: *p* = 0.0143) which then decreased at 2–3 wpi (Figure 3G left panel; *N* = 4 animals, Tukey’s multiple comparison test: *p* = 0.0079) and further decreased at 10–12 wpi (Figure 3G left panel; *N* = 5 animals, Tukey’s multiple comparison test: *p* = 0.0002). The same pattern was observed in the trained animals where there was an increase at 6 dpi compared to sham (Figure 3G right panel; *N* = 10 in sham and *N* = 4 animals at 6 dpi, Tukey’s multiple comparison test: *p* = 0.0059) and then later decreased at 4–6 wpi (Figure 3G right panel; *N* = 6 animals, Tukey’s multiple comparison test: *p* = 0.0103). A pairwise comparison between the untrained and trained groups showed a significant increase in the level of presynaptic inhibition at 2–3 wpi in the mice that received treadmill training (Figure 3I; *t*-tests with Bonferroni’s multiple-comparisons correction: *p* = 0.014).

The size of GABApre terminals was measured in dI3 INs (Figure 3C; *n* = 359–521 boutons in untrained animals and *n* = 453–636 boutons in trained animals; one-factor ANOVA, *p* < 0.0001 for untrained and trained animals) and MNs (Figure 3H; *n* = 1071–1326 boutons in untrained animals and *n* = 1301–1719 boutons in trained animals; one-factor ANOVA, *p* < 0.0001 for untrained and trained animals). The GABApre terminals on VGLUT1^+^ inputs to dI3 INs or MNs in sham (dI3 INs: mean = 0.8 ± 0.2 μm; MNs: mean = 0.8 ± 0.2 μm) were smaller in SCI mice, where the mean ranged between 0.6 and 0.7 μm for both dI3 INs and MNs. Pairwise comparisons of GABApre terminal size on VGLUT1^+^ boutons in dI3 INs and MNs from trained vs. untrained mice showed differences at all injury time points (Figures 3E, J; *t*-tests with Bonferroni’s multiple-comparisons correction, *p* < 0.0001 for all time points).

Overall, levels of presynaptic inhibition to VGLUT1^+^ inputs to dI3 INs in untrained animals and MNs in untrained and trained animals showed a pattern of short-term increases compared to sham levels, followed by decreases though never to levels below sham levels. There were no detectable changes in presynaptic inhibition to VGLUT1^+^ inputs to dI3 INs in trained animals. Training increased presynaptic inhibition to VGLUT1^+^ inputs to MNs at 2–3 wpi.

### Comparing between upper and lumbar dI3 INs and MNs

We compared changes in synaptic inputs to upper (L1–L3) vs. lower (L4–L6) lumbar dI3 INs and MNs (Supplementary Figures 1, 2). Most of the changes observed in the overall population of lumbar dI3 INs and MNs were observed in the upper and lower lumbar subsets of dI3 INs and MNs. We did notice that changes in the number of VGLUT1^+^ boutons after SCI were only observed in lower lumbar dI3 INs. At the same time, the decrease in presynaptic inhibition after SCI that was seen in all MNs was absent in lower lumbar MNs.

### Sex-related differences in the changes in synaptic inputs to dI3 INs and MNs after SCI

We have also performed an analysis of possible sex-related differences in the changes in synaptic inputs to dI3 INs and MNs after SCI. A two-way ANOVA for injury time-point and sex was performed for all of the changes in synaptic inputs to dI3 INs and MNs described above. Sex was a factor for the number of VGLUT2^+^ inputs to dI3 INs, the size of VGLUT1^+^ boutons to dI3 INs and MNs, and the size of GABApre terminals contacting VGLUT1^+^ inputs to dI3 INs (Supplementary Figure 3).

### Effects of stepping function on levels of synaptic inputs to dI3 INs and MNs

**FIGURE 4:**
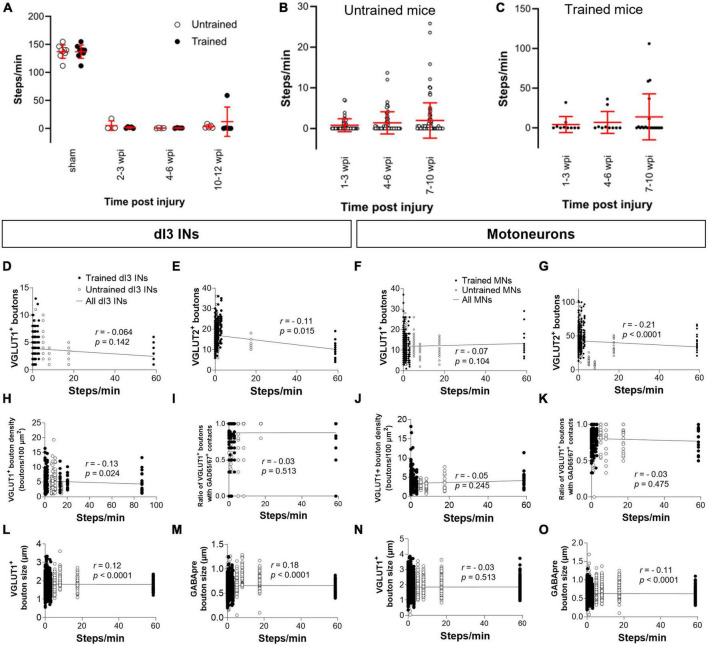
Analysis of stepping function without weight support after SCI and measures of synaptic inputs **(A)** Number of steps per minute at the experimental endpoint or in sham animals. **(B)** The number of steps per minute at different time points in untrained or in **(C)** trained animals with 10–12 wpi experimental endpoint. The number of VGLUT1^+^ boutons, number of VGLUT2^+^ boutons, density of VGLUT1^+^ boutons, percent of VGLUT1^+^ boutons with GAD65/67^+^ contacts, size of VGLUT1^+^ boutons, and size of VGLUT1^+^ boutons to lumbar dI3 INs [**(D,E,H,I,L,M)**, respectively] and MNs [**(F,G,J,K,N,O)**, respectively] to MNs in relation to the number of steps per minute made by the SCI mice from which each neuron came from in their last testing session. Circles denote counts for individual cells. Red lines denote ± SD. Black lines in **(D–O)** indicate linear regression.

Each animal in our experimental cohort underwent weekly testing for stepping function on a treadmill without any weight support provided. The number of steps performed per minute was estimated using DeepLabCut for automated hindlimb joint detection. We found no differences in the number of steps between trained and untrained animals at their experimental endpoints (Figure 4A; one-factor ANOVA, *p* = 0.1950). Trained and untrained SCI animals made fewer steps than sham animals at their respective experimental endpoints (Figure 4A; Tukey’s multiple comparison test: *p* < 0.0001). To determine whether there was any improvement in stepping function with time post-injury, we compared the stepping function at different time points in animals whose experimental endpoint was 10–12 wpi. While some animals had their highest stepping function between 7 and 10 wpi, statistical testing failed to detect any differences in stepping function between the different time points (Figures 4B, C; one-factor ANOVA, *p* = 0.1395 for untrained animals, *p* = 0.2885 for trained animals).

To determine whether there was any correlation between stepping function and levels of synaptic inputs, we performed a correlation analysis between the number of various synaptic inputs with the number of steps per minute of mice at the experimental endpoint using the non-parametric Spearman Correlation test. Correlations could not be detected between the number of VGLUT1^+^ inputs, or presynaptic inhibition of VGLUT1^+^ inputs to dI3 INs (Figures 4D, I) or MNs (Figures 4F, K) and stepping function. On the other hand, there was a negative correlation between the number of VGLUT2^+^ inputs to dI3 INs (Figure 4E) and MNs (Figure 4G) and stepping function. There were no correlations between VGLUT1^+^ bouton density on dI3 INs or MNs and stepping function (Figures 4H, J). We also tested for possible correlations with VGLUT1^+^ bouton size and stepping function and found weak positive correlations for both dI3 INs and MNs (Figures 4L, N). Weak positive correlations for both dI3 INs and MNs was also found between GABApre bouton size and stepping function (Figures 4M, O).

**FIGURE 5:**
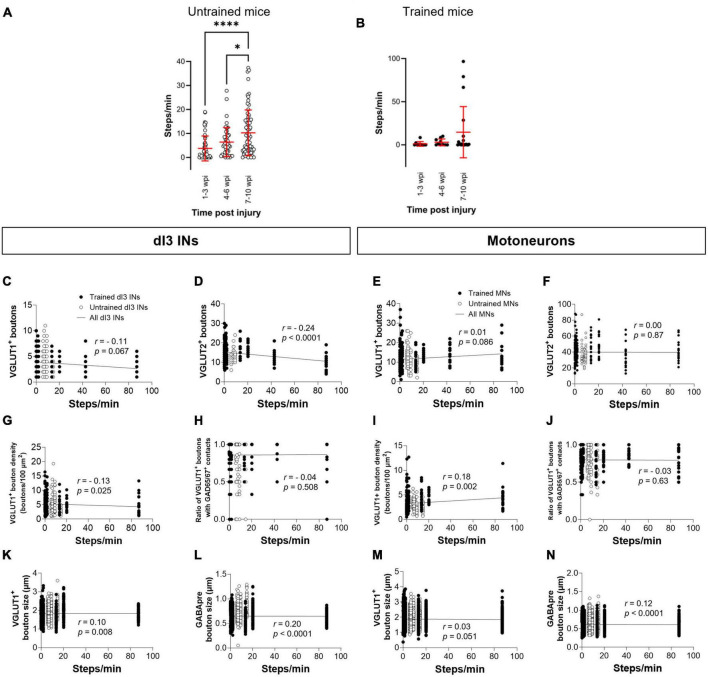
Analysis of stepping function with body weight support after SCI and measures of synaptic inputs **(A)** The number of steps per minute at different time points in untrained or in **(B)** trained animals with 10–12 wpi experimental endpoint. The number of VGLUT1^+^ boutons, number of VGLUT2^+^ boutons, density of VGLUT1^+^ boutons, percent of VGLUT1^+^ boutons with GAD65/67^+^ contacts, size of VGLUT1^+^ boutons, and size of VGLUT1^+^ boutons to lumbar dI3 INs [**(C,D,G,H,K,L)**, respectively] and MNs [**(E,F,I,J,M,N)**, respectively] to MNs in relation to the number of steps per minute made by the SCI mice from which each neuron came from in their last testing session. Circles denote counts for individual cells. Red lines denote ± SD. Black lines in **(C–N)** indicate linear regression. *p ≤ 0.05, ****p ≤ 0.0001 (Tukey’s multiple comparison test following one-factor ANOVA).

Animals with experiment endpoints at 10–12 wpi also had an additional testing session where they walked with manual body weight support. We compared the stepping function with body weight support at different time points. Stepping function with body weight support improved with time post-injury in untrained animals (Figure 5A; *N* = 5 animals; one-factor ANOVA, *p* < 0.001) from 4–6 to 7–10 wpi (Figure 5A; Tukey’s multiple comparison test: *p* = 0.0186) such that stepping function between 1–3 and 7–10 wpi was also significantly different (Figure 5A; Tukey’s multiple comparison test: *p* < 0.0001). Due to lighting issues, the number of steps with body weight support for the trained animals was counted manually rather than using DeepLabCut. In the trained animals, stepping function did not improve with time post-injury (Figure 5B; *N* = 5 animals; one-factor ANOVA, *p* = 0.1869). Correlations could not be detected between levels of VGLUT1^+^ inputs or presynaptic inhibition of VGLUT1^+^ inputs to dI3 INs (Figures 5C, H) or MNs (Figures 5E, J) and stepping function in animals with body weight support. While there was a negative correlation between VGLUT2^+^ inputs to dI3 INs and stepping function in animals with body weight support (Figure 5D), no correlation was detected for VGLUT2^+^ inputs to MNs (Figure 5F). There was a negative correlation between VGLUT1^+^ bouton density in dI3 INs and stepping function with body weight support (Figure 5G). In contrast, there was a positive correlation VGLUT1^+^ bouton density in MNs and stepping function with body weight support was found (Figure 5I). Just as for stepping without weight support, we found weak positive correlations with VGLUT1^+^ bouton size as well as GABApre bouton size and stepping function with body weight support for dI3 INs (Figures 5K, L), and between GABApre bouton size and stepping function with body weight support for MNs (Figures 5M, N).

## Discussion

Injury to the spinal cord disrupts the descending fibers from the brain onto the spinal cord. However, spinal circuits caudal to the spinal cord lesions regain some capacity to generate locomotor activity. Locomotor activity after spinalization can be readily expressed on a treadmill in some mammals. Whether the recovery of locomotor function after SCI relies upon changes in the connectivity of spinal circuits is unknown. In the present study, we investigated changes in synaptic inputs to dI3 INs and MNs with a focus on sensorimotor integration.

### Timecourse of changes in synaptic inputs to dI3 INs and MNs

We first investigated possible changes in sensory afferent inputs onto dI3 INs and MNs following SCI and in response to treadmill training. We quantified the number of VGLUT1^+^ boutons on the soma and perisomatic dendrites of the dI3 INs and MNs. Our data reveal decreases (in untrained animals) or short-term increases (in trained animals) at several time points in the number of VGLUT1^+^ boutons to dI3 INs. Normalizing the number of VGLUT1^+^ inputs to dI3 INs by an estimate of soma area suggests a decrease in VGLUT1^+^ bouton density in dI3 INs from untrained animals. MNs did not show any changes in the number of VGLUT1^+^ inputs, but when normalized to an estimate of soma area, there was an increase at longer time points after injury. We also observed a slight decrease in the size of VGLUT1^+^ boutons to dI3 INs and MNs.

Other studies investigating changes in VGLUT1^+^ boutons in the spinal cord have reported either decreases or a lack of change in these inputs after spinal cord injury. Khalki et al. (2018) observed a lower density of VGLUT1^+^ terminals on the surface of gastrocnemius (GS) and tibialis anterior (TA) MNs in untrained adult rats with a complete mid-thoracic transection compared to intact animals. No differences were observed in the density of VGLUT1^+^ terminals between trained SCI and intact animals (Khalki et al., 2018). Following complete sacral transection in the adult rat, Kapitza et al. (2012) observed decreases in VGLUT1^+^ bouton density in medial lamina VII at 12 wpi but not earlier and decreases at 4 wpi but not 1 or 12 wpi in lateral laminae VII. No changes were observed in lamina IX of S3–S4 segments (Kapitza et al., 2012). Thus, our observations in dI3 INs but not in MNs are consistent with previous reports of the intact or decreased density of VGLUT1^+^ boutons after spinal cord injury. Khalki et al. (2018) observed an increase in the size of VGLUT1^+^ boutons to motoneurons whereas we observed a minimal but significant decrease in the size of these inputs to both dI3 INs and MNs.

We then looked at changes in central excitatory inputs onto dI3 INs and MNs using VGLUT2 as a marker for intact propriospinal interneuron inputs assuming a lack of descending supraspinal inputs due to the spinal transection. We observed a significant increase in VGLUT2^+^ inputs to dI3 INs at 2–3 wpi, which decreased in the time points after 4–6 wpi in trained animals. In the untrained animals, VGLUT2^+^ inputs to dI3 INs decreased significantly at time points after 4–6 wpi. We also observed a significant decrease in VGLUT2^+^ inputs to MNs in both untrained and trained animals at 6 dpi compared to sham levels. Subsequently, there were increases in both groups at 2–3 wpi followed by decreases after 4–6 wpi such that levels at 10–12 wpi were below sham levels. A similar reduction by 20% of VGLUT2^+^ inputs in the spinal cord was observed 1 week after S2 transection in adult rats, and this reduction remained up to 12 weeks post-injury (Kapitza et al., 2012).

In addition to changes in excitatory inputs after SCI, the loss of supraspinal descending inputs also alters inhibitory synaptic transmission. Transmission of sensory excitation to spinal circuits can be regulated by GABAergic transmission onto the central terminals of sensory afferents (Rudomin, 1999; Betley et al., 2009; Hari et al., 2022). We looked at changes in the presynaptic inhibition of VGLUT1^+^ sensory afferents onto dI3 INs and MNs. Changes in presynaptic inhibition of VGLUT1^+^ boutons to dI3 INs and MNs in SCI mice were modest and occurred in a limited number of time points studied. There was a significant increase in presynaptic inhibition of VGLUT1^+^ inputs to dI3 INs in the untrained mice at 2–3 wpi compared to sham, which was followed by a decrease at 10–12 wpi. In the MNs, a significant increase in presynaptic inhibition of VGLUT1^+^ inputs in both untrained and trained mice was observed at 6 dpi. However, this increase was lost afterward, with presynaptic inhibition of VGLUT1^+^ inputs decreasing in the untrained mice at 2–3 wpi and in the trained mice at 4–6 wpi. Levels of presynaptic inhibition of VGLUT1^+^ inputs were never observed to fall below sham levels for dI3 INs and MNs in untrained or trained animals.

Khalki et al. (2018) reported an increase in the density of presynaptic GABAergic terminals onto VGLUT1^+^ terminals to GS and TA MNs, in addition to increases in inhibitory GABAergic and glycinergic inputs onto cell bodies of GS and TA MNs of the trained and untrained SCI rats compared to intact. Interestingly, the size of the presynaptic inhibitory boutons was increased in untrained SCI rats but reduced in trained SCI rats, suggesting that the increased density of presynaptic inhibitory terminals was due to the sprouting of existing axons conveying presynaptic inhibition (Khalki et al., 2018). While we observed a decrease in the size of GABApre terminals on dI3 INs and MNs in both untrained and trained SCI mice, the decrease was greater in trained mice, perhaps due to more sprouting in the latter population. A different study reported a sustained decrease in GABAergic terminals contacting VGLUT1^+^ boutons in the sacral spinal cord after sacral transection (Kapitza et al., 2012).

The changes in the overall levels of GAD65 or GAD67 expression in the spinal cord after SCI have also been studied. Adult cats with complete thoracic transection exhibit increased GAD67 puncta in lamina IX (Tillakaratne et al., 2002). There were also increased levels of GAD67 expression in the ventral and dorsal horns of L5–L7 after thoracic transection, which decreased to control levels with step training (Tillakaratne et al., 2002). No increases in GAD65 levels were observed after thoracic transection in the adult cat (Tillakaratne et al., 2000). The balance of inhibitory to excitatory synaptic inputs to motoneurons shifts toward inhibition in neonatal rats with a midthoracic spinal cord transection at postnatal day 5. This balance seems to be restored to intact levels by locomotor training (Ichiyama et al., 2011). Reduced GAD65 and GAD67 immunoreactivity was observed in the L4–L5 dorsal horn of mice compared to sham at 6 wpi following a moderate contusion injury to T11 of GAD67:GFP mice (Meisner et al., 2010). Decreased levels of GAD65 and 67 expressions were also observed in the rat dorsal horn following contusion at T10 (Li et al., 2020).

We did not investigate GABA-mediated presynaptic inhibition of VGLUT2 + afferents in the spinal cord. Central terminals of Aδ-fibers can be gated by GABA-mediated presynaptic inhibition (Zimmerman et al., 2019). dI3 INs may receive direct inputs from these fibers based on electrophysiological recordings (Bui et al., 2013), but this sensory connection has not been confirmed anatomically. Presynaptic inhibition of VGLUT2 + terminals from spinal or supraspinal neurons to motor circuits has not been reported to the best of our knowledge.

### Possible mechanisms underlying plasticity

Losses of VGLUT1^+^ and VGLUT2^+^ inputs shortly after SCI may reflect the severing of descending supraspinal and propriospinal inputs to circuits below the lesion. However, we did not observe any loss of VGLUT1^+^ inputs to dI3 INs and MNs shortly after SCI, and short-term losses in VGLUT2^+^ inputs were only observed to MNs and not dI3 INs. This absence of immediate loss of VGLUT1^+^ and VGLUT2^+^ inputs could reflect a paucity of descending supraspinal or propriospinal inputs to dI3 INs or could indicate these connections are made to the dendritic regions that were not sampled in our image analysis.

Some studies have linked changes in neurotransmitter expression after SCI to neurotrophin signaling. For example, BDNF has been linked to several signal transduction cascades that promote neurogenesis, axonal sprouting, and neuronal survival (Friedman et al., 1995; Arvanian and Mendell, 2001; Koda et al., 2002; Nakajima et al., 2010; Keefe et al., 2017). BDNF levels may decrease shortly after spinal cord injury (Gulino et al., 2004; Gómez-Pinilla et al., 2004). However, rehabilitation programs, such as treadmill training or cycling, may reactivate silent spinal circuits, which could increase the level of BDNF in the spinal cord (Gómez-Pinilla et al., 2002; Ying et al., 2005; Côté et al., 2011; Boyce et al., 2012).

BDNF signaling can shape presynaptic inhibition of sensory inputs in the spinal cord. Activity-based exercise training seems to increase the synthesis of GAD65 and 67, and this mechanism may be related to exercise-induced BDNF synthesis and TrKB signaling activation in the spinal cord (Keeler et al., 2012; Huang et al., 2017; Leech and Hornby, 2017). Furthermore, the release of glutamate from sensory terminals seems to affect the differentiation and function of GABAergic terminals mediating presynaptic inhibition of sensory afferents via metabotropic glutamate receptor mGluR1 expressed on the GABAergic terminals (Mende et al., 2016). Since the expression of GAD65 is regulated by autocrine influence of BDNF on sensory terminals (Betley et al., 2009), the combined reduction of glutamate release from sensory neurons and BDNF that could result from loss of locomotor activity after spinal cord injury could theoretically drive the loss of GAD65 expression in spinal circuits. However, we failed to observe large changes in GAD65 expressing boutons on VGLUT1^+^ terminals to dI3 INs or MNs.

Recent findings have uncovered a novel mechanism underlying changes in neurotransmitter levels in the injured spinal cord. A subset of VGLUT2^+^ interneurons, including dI3 INs, were found to switch neurotransmitter phenotype from excitatory glutamatergic to inhibitory GABAergic synapses contacting MNs after a complete T10 transection in adult mice (Bertels et al., 2022). This neurotransmitter phenotype switching could also explain some of the reported loss of VGLUT2^+^ boutons or rise in GAD65/67 + boutons in the spinal cord observed after SCI (Tillakaratne et al., 2002; Kapitza et al., 2012; Khalki et al., 2018).

### Training vs. non-training

We investigated for possible differences in synaptic inputs to dI3 INs and MNs from SCI animals with or without treadmill training. We observed more VGLUT1^+^ boutons onto the surface of dI3 INs at 2–3 wpi in trained animals than untrained animals. However, no difference was observed in the level of VGLUT1^+^ boutons on MNs between the trained and untrained animals, nor were there differences in VGLUT1^+^ boutons to MNs from SCI or sham groups. Similar to VGLUT1^+^ inputs, we found more VGLUT2^+^ boutons onto dI3 INs of trained mice compared to untrained at 2–3 wpi and in MNs of trained mice at 2–3 wpi compared to untrained animals. On the other hand, we did not observe any effect of training on the level of presynaptic inhibition in dI3 INs. However, the trained mice had more presynaptic inhibition of VGLUT1^+^ inputs to MNs at 2–3 wpi than untrained animals. We found only modest differences in synaptic inputs to dI3 INs and MNs between SCI animals with or without treadmill training.

Our results contrast with Khalki et al. (2018), who showed that MNs from SCI-trained mice had a higher density of VGLUT1^+^ boutons compared to MNs from both intact and untrained SCI mice. Their study also reported that VGLUT1^+^ terminals on MNs were larger in both untrained and trained mice compared to intact mice (Khalki et al., 2018), something that we have not quantified. Step training has been suggested to restore levels of the γ2 subunit of GABA_A_ receptors in motoneurons of rats 3 months after complete midthoracic transections at neonatal stages (Khristy et al., 2009). On the other hand, Cantoria et al. (2011) failed to detect any significant difference in the densities of VGLUT1^+^ and glycinergic GLYT2 inputs within the spinal cord of trained and untrained neonatal rats with complete transection (Cantoria et al., 2011).

### Methodological considerations

Differences in how activity-based training influences the plasticity of spinal circuits after SCI may be related to variations in how SCI animals are trained. Our training regimen was comparatively lighter in frequency and duration than in several other studies, which may have led to the lack of significant improvements in stepping function in trained vs. untrained animals. The lighter locomotor training regimen could have limited the release of any signaling molecule, such as neurotrophins, and any remodeling of spinal circuits. Furthermore, we did not consider the administration of supplementary interventions to improve locomotor recovery, such as the administration of pharmacological neuromodulation (Khalki et al., 2018) or electrical stimulation (Lavrov et al., 2008; Taccola et al., 2018; Gerasimenko et al., 2019; Zhang et al., 2021; Kathe et al., 2022). However, recovery of locomotor function could also be associated with the levels of home-cage activity between training sessions (Caudle et al., 2011; Torres-Espín et al., 2018). The lack of monitoring of this activity in our study and others makes it harder to truly associate training with recovery (Torres-Espín et al., 2018)Click or tap here to enter text.

Several other factors may explain the discrepancies between our findings and those reported by other studies. These factors include the differences in animal model (species and age at which the injury occurs), injury type/severity, time after injury at which observations are made, the nature of any intervention (activity-based, neuromodulation, electrical), and the region of the spinal cord and the compartments of the neurons where the levels of VGLUT1^+^, VGLUT2^+^, and GAD65/67^+^ inputs were quantified. For example, we investigated unidentified lumbar motoneurons located in L1-L5 in adult mice, while others have sampled from specific motor pools (Khalki et al., 2018) or motoneurons in sacral segments (Kapitza et al., 2012). Furthermore, we studied synaptic inputs made onto the soma of the motoneurons and the proximal 100 μm of the dendritic tree, while others sampled from further out into the dendritic tree. Thus, our conclusions of limited changes to sensory and central glutamatergic inputs and presynaptic inhibition to dI3 INs and MNs may apply only to the somatic and perisomatic regions of these neuron populations. Finally, the sex of the animals studied could also be a factor. For instance, in most experiments, only female rats (Kapitza et al., 2012; Khalki et al., 2018) or cats (Tillakaratne et al., 2002) were used, but we used both male and female mice. Whether there are sex differences in remodeling of spinal locomotor circuits remains to be investigated.

Overall, our results suggest modest changes in synaptic inputs related to sensorimotor integration to MNs and dI3 INs after spinal cord injury. Restoring motoneuron activity is evidently beneficial to the recovery of motor function after SCI. Silencing dI3 INs impairs locomotor recovery after SCI (Bui et al., 2016). Our data do not provide strong evidence that certain levels of synaptic inputs from any specific circuits must be prioritized in restoring movements after SCI. In fact, a negative correlation was found between central excitatory inputs and stepping function. It is unclear why the loss of excitation would improve stepping function after injury. We conclude instead that the modest changes in synaptic inputs to MNs and dI3 INs suggest that those circuits are available to be recruited if this recruitment is eventually found to be beneficial to the recovery of locomotor function.

## Data availability statement

The raw data supporting the conclusions of this article will be made available by the authors, without undue reservation.

## Ethics statement

The animal study was reviewed and approved by the University of Ottawa Animal Care Committee.

## Author contributions

SG contributed to the data collection, analysis, figures preparation, and writing of the manuscript. SS and ET quantified stepping function using DeepLabCut. TB contributed to the conceptualization, supervision, data analysis, and writing of the manuscript. All authors contributed to the article and approved the submitted version.

## References

[B1] Alvarez F. J. Villalba R. M. Zerda R. Schneider S. P. (2004). Vesicular glutamate transporters in the spinal cord, with special reference to sensory primary afferent synapses. *J. Comp. Neurol.* 472 257–280. 10.1002/cne.20012 15065123

[B2] Arvanian V. L. Mendell L. M. (2001). Acute modulation of synaptic transmission to motoneurons by BDNF in the neonatal rat spinal cord. *Eur. J. Neurosci.* 14 1800–1808. 10.1046/j.0953-816x.2001.01811.x 11860475

[B3] Asboth L. Friedli L. Beauparlant J. Martinez-Gonzalez C. Anil S. Rey E. (2018). Cortico–reticulo–spinal circuit reorganization enables functional recovery after severe spinal cord contusion. *Nat. Neurosci.* 21 576–588. 10.1038/s41593-018-0093-5 29556028

[B4] Bareyre F. M. (2008). Neuronal repair and replacement in spinal cord injury. *J. Neurol. Sci.* 265 63–72. 17568612 10.1016/j.jns.2007.05.004

[B5] Bareyre F. M. Kerschensteiner M. Raineteau O. Mettenleiter T. C. Weinmann O. Schwab M. E. (2004). The injured spinal cord spontaneously forms a new intraspinal circuit in adult rats. *Nat. Neurosci.* 7 269–277. 10.1038/nn1195 14966523

[B6] Bertels H. Vicente-Ortiz G. el Kanbi K. Takeoka A. (2022). Neurotransmitter phenotype switching by spinal excitatory interneurons regulates locomotor recovery after spinal cord injury. *Nat. Neurosci.* 25 617–629. 10.1038/s41593-022-01067-9 35524138 PMC9076533

[B7] Betley J. N. Wright C. V. E. Kawaguchi Y. Erdélyi F. Szabó G. Jessell T. M. (2009). Stringent specificity in the construction of a GABAergic presynaptic inhibitory circuit. *Cell* 139 161–174. 10.1016/j.cell.2009.08.027 19804761 PMC2812434

[B8] Bouyer L. J. G. Rossignol S. (2003). Contribution of cutaneous inputs from the hindpaw to the control of locomotion. II. spinal cats. *J. Neurophysiol.* 90 3640–3653. 10.1152/jn.00497.2003 12944535

[B9] Boyce V. S. Park J. Gage F. H. Mendell L. M. (2012). Differential effects of brain-derived neurotrophic factor and neurotrophin-3 on hindlimb function in paraplegic rats. *Eur. J. Neurosci.* 35 221–232. 10.1111/j.1460-9568.2011.07950.x 22211901 PMC3509221

[B10] Brownstone R. M. Bui T. V. (2010). Spinal interneurons providing input to the final common path during locomotion. *Prog. Brain Res.* 187 81–95. 10.1016/B978-0-444-53613-6.00006-X 21111202 PMC3150186

[B11] Bui T. V. Akay T. Loubani O. Hnasko T. S. Jessell T. M. Brownstone R. M. (2013). Circuits for grasping: spinal dI3 interneurons mediate cutaneous control of motor behavior. *Neuron* 78 191–204. 10.1016/j.neuron.2013.02.007 23583114 PMC4535710

[B12] Bui T. V. Stifani N. Akay T. Brownstone R. M. (2016). Spinal microcircuits comprising dI3 interneurons are necessary for motor functional recovery following spinal cord transection. *eLife* 5:e21715. 10.7554/eLife.21715 27977000 PMC5218533

[B13] Cantoria M. J. See P. A. Singh H. De Leon R. D. (2011). *Development/plasticity/repair adaptations in glutamate and glycine content within the lumbar spinal cord are associated with the generation of novel gait patterns in rats following neonatal spinal cord transection.* Available online at: 10.1523/JNEUROSCI.3499-11.2011 PMC3268368 22171058

[B14] Capelli P. Pivetta C. Esposito M. S. Arber S. (2017). Locomotor speed control circuits in the caudal brainstem. *Nature* 551 373–377. 29059682 10.1038/nature24064

[B15] Caudle K. L. Brown E. H. Shum-Siu A. Burke D. A. Magnuson T. S. G. Voor M. J. (2011). Hindlimb immobilization in a wheelchair alters functional recovery following contusive spinal cord injury in the adult rat. *Neurorehabil. Neural Repair.* 25 729–739. 10.1177/1545968311407519 21697451 PMC4419333

[B16] Côté M. P. Azzam G. A. Lemay M. A. Zhukareva V. Houlé J. D. (2011). Activity-dependent increase in neurotrophic factors is associated with an enhanced modulation of spinal reflexes after spinal cord injury. *J. Neurotrauma* 28 299–309. 10.1089/neu.2010.1594 21083432 PMC3037803

[B17] Côté M.-P. Detloff M. R. Wade R. E. Lemay M. A. Houlé J. D. Houlé J. D. (2012). Plasticity in ascending long propriospinal and descending supraspinal pathways in chronic cervical spinal cord injured rats. *Front. Physiol.* 3:330. 10.3389/fphys.2012.00330 22934078 PMC3429098

[B18] Courtine G. Gerasimenko Y. van den Brand R. Yew A. Musienko P. Zhong H. (2009). Transformation of nonfunctional spinal circuits into functional states after the loss of brain input. *Nat. Neurosci.* 12 1333–1342. 10.1038/nn.2401 19767747 PMC2828944

[B19] Courtine G. Song B. Roy R. R. Zhong H. Herrmann J. E. Ao Y. (2008). Recovery of supraspinal control of stepping via indirect propriospinal relay connections after spinal cord injury. *Nat. Med.* 14 69–74. 10.1038/nm1682 18157143 PMC2916740

[B20] Cowley K. C. MacNeil B. J. Chopek J. W. Sutherland S. Schmidt B. J. (2015). Neurochemical excitation of thoracic propriospinal neurons improves hindlimb stepping in adult rats with spinal cord lesions. *Exp. Neurol.* 264 174–187. 10.1016/j.expneurol.2014.12.006 25527257

[B21] Cummings J. P. Bernstein D. R. Stelzner D. J. (1981). Further evidence that sparing of function after spinal cord transection in the neonatal rat is not due to axonal generation or regeneration. *Exp. Neurol.* 74 615–620. 6170524 10.1016/0014-4886(81)90196-5

[B22] Edgerton V. R. Courtine G. Gerasimenko Y. P. Lavrov I. Ichiyama R. M. Fong A. J. (2007). Training locomotor networks. *Brain Res. Rev.* 57 241–254. 10.1016/j.brainresrev.2007.09.002 18022244 PMC2288528

[B23] Edgerton V. R. Tillakaratne N. J. K. Bigbee A. J. de Leon R. D. Roy R. R. (2004). Plasticity of the spinal neural circuitry after injury. *Annu. Rev. Neurosci.* 27 145–167. 15217329 10.1146/annurev.neuro.27.070203.144308

[B24] Erlander M. G. Tobin A. J. (1991). The structural and functional heterogeneity of glutamic acid decarboxylase: a review. *Neurochem. Res.* 16 215–226. 1780024 10.1007/BF00966084

[B25] Flynn J. R. Graham B. A. Galea M. P. Callister R. J. (2011). The role of propriospinal interneurons in recovery from spinal cord injury. *Neuropharmacology* 60 809–822. 21251920 10.1016/j.neuropharm.2011.01.016

[B26] Friedman B. Kleinfeld D. Ip N. Y. Verge V. M. K. Moulton R. Boland P. (1995). BDNF and NT-4/5 exert neurotrophic influences on injured adult spinal motor neurons. *J. Neurosci.* 15 1044–1056. 10.1523/JNEUROSCI.15-02-01044.1995 7869082 PMC6577802

[B27] Frigon A. Rossignol S. (2006). Functional plasticity following spinal cord lesions. *Prog. Brain Res.* 157 231–260. 17167915 10.1016/s0079-6123(06)57016-5

[B28] Frigon A. Rossignol S. (2008). Adaptive changes of the locomotor pattern and cutaneous reflexes during locomotion studied in the same cats before and after spinalization. *J. Physiol.* 586 2927–2945. 10.1113/jphysiol.2008.152488 18420704 PMC2517203

[B29] Gerasimenko Y. Preston C. Zhong H. Roy R. R. Edgerton V. R. Shah P. K. (2019). Rostral lumbar segments are the key controllers of hindlimb locomotor rhythmicity in the adult spinal rat. *J. Neurophysiol.* 122 585–600. 10.1152/jn.00810.2018 30943092 PMC6734399

[B30] Gerasimenko Y. P. Makarovskii A. N. Nikitin O. A. (2002). Control of locomotor activity in humans and animals in the absence of supraspinal influences. *Neurosci. Behav. Physiol.* 32 417–423. 12243263 10.1023/a:1015836428932

[B31] Gómez-Pinilla F. Ying Z. Roy R. R. Hodgson J. Edgerton V. R. Fernando G.-P. (2004). Afferent input modulates neurotrophins and synaptic plasticity in the spinal cord. *J. Neurophysiol.* 92 3423–3432. 15548637 10.1152/jn.00432.2004

[B32] Gómez-Pinilla F. Ying Z. Roy R. R. Molteni R. Edgerton V. R. (2002). Voluntary exercise induces a BDNF-mediated mechanism that promotes neuroplasticity. *J. Neurophysiol.* 88 2187–2195. 10.1152/jn.00152.2002 12424260

[B33] Gulino R. Lombardo S. A. Casabona A. Leanza G. Perciavalle V. (2004). Levels of brain-derived neurotrophic factor and neurotrophin-4 in lumbar motoneurons after low-thoracic spinal cord hemisection. *Brain Res.* 1013 174–181. 10.1016/j.brainres.2004.03.055 15193526

[B34] Hari K. Lucas-Osma A. M. Metz K. Lin S. Pardell N. Roszko D. A. (2022). GABA facilitates spike propagation through branch points of sensory axons in the spinal cord. *Nat. Neurosci.* 25 1288–1299. 36163283 10.1038/s41593-022-01162-x PMC10042549

[B35] Huang Y. J. Lee K. H. Grau J. W. (2017). Complete spinal cord injury (SCI) transforms how brain derived neurotrophic factor (BDNF) affects nociceptive sensitization. *Exp. Neurol.* 288 38–50. 10.1016/j.expneurol.2016.11.001 27818188

[B36] Hughes D. I. Mackie M. Nagy G. G. Riddell J. S. Maxwell D. J. Szabó G. (2005). P boutons in lamina IX of the rodent spinal cord express high levels of glutamic acid decarboxylase-65 and originate from cells in deep medial dorsal horn. *Proc. Natl. Acad. Sci. USA* 102, 9038–9043. 10.1073/PNAS.0503646102 15947074 PMC1157050

[B37] Ichiyama R. M. Broman J. Roy R. R. Zhong H. Edgerton V. R. Havton L. A. (2011). Locomotor training maintains normal inhibitory influence on both alpha- and gamma-motoneurons after neonatal spinal cord transection. *J. Neurosci.* 31 26–33. 10.1523/JNEUROSCI.6433-09.2011 21209186 PMC3036743

[B38] Kapitza S. Zörner B. Weinmann O. Bolliger M. Filli L. Dietz V. (2012). Tail spasms in rat spinal cord injury: changes in interneuronal connectivity. *Exp. Neurol.* 236 179–189. 10.1016/j.expneurol.2012.04.023 22569103

[B39] Kathe C. Skinnider M. A. Hutson T. H. Regazzi N. Gautier M. Demesmaeker R. (2022). The neurons that restore walking after paralysis. *Nature* 611 540–547. 36352232 10.1038/s41586-022-05385-7 PMC9668750

[B40] Keefe K. M. Sheikh I. S. Smith G. M. (2017). Targeting neurotrophins to specific populations of neurons: NGF, BDNF, and NT-3 and their relevance for treatment of spinal cord injury. *Int. J. Mol. Sci.* 18:548. 10.3390/ijms18030548 28273811 PMC5372564

[B41] Keeler B. E. Liu G. Siegfried R. N. Zhukareva V. Murray M. Houlé J. D. (2012). Acute and prolonged hindlimb exercise elicits different gene expression in motoneurons than sensory neurons after spinal cord injury. *Brain Res.* 1438 8–21. 10.1016/j.brainres.2011.12.015 22244304 PMC3273584

[B42] Khalki L. Sadlaoud K. Lerond J. Coq J. O. Brezun J. M. Vinay L. (2018). Changes in innervation of lumbar motoneurons and organization of premotor network following training of transected adult rats. *Exp. Neurol.* 299 1–14. 10.1016/j.expneurol.2017.09.002 28917641

[B43] Khristy W. Ali N. J. Bravo A. B. de Leon R. Roy R. R. Zhong H. (2009). Changes in GABAA receptor subunit gamma 2 in extensor and flexor motoneurons and astrocytes after spinal cord transection and motor training. *Brain Res.* 1273 9–17. 10.1016/j.brainres.2009.03.060 19358834 PMC2700157

[B44] Koda M. Murakami M. Ino H. Yoshinaga K. Ikeda O. Hashimoto M. (2002). Brain-derived neurotrophic factor suppresses delayed apoptosis of oligodendrocytes after spinal cord injury in rats. *J. Neurotrauma* 19 777–785. 10.1089/08977150260139147 12165137

[B45] Laliberte A. M. Farah C. Steiner K. R. Tariq O. Bui T. V. (2022). Changes in sensorimotor connectivity to dI3 interneurons in relation to the postnatal maturation of grasping. *Front. Neural Circuits* 15:768235. 10.3389/fncir.2021.768235 35153680 PMC8828486

[B46] Laliberte A. M. Goltash S. Lalonde N. R. Bui T. V. (2019). Propriospinal neurons: essential elements of locomotor control in the intact and possibly the injured spinal cord. *Front. Cell Neurosci.* 13:512. 10.3389/fncel.2019.00512 31798419 PMC6874159

[B47] Lavrov I. Courtine G. Dy C. J. van den Brand R. Fong A. J. Gerasimenko Y. (2008). Facilitation of stepping with epidural stimulation in spinal rats: role of sensory input. *J. Neurosci.* 28 7774–7780. 10.1523/JNEUROSCI.1069-08.2008 18667609 PMC2897701

[B48] Leech K. A. Hornby T. G. (2017). High-intensity locomotor exercise increases brain-derived neurotrophic factor in individuals with incomplete spinal cord injury. *J. Neurotrauma* 34 1240–1248. 10.1089/neu.2016.4532 27526567 PMC5359683

[B49] Li X. Wang Q. Ding J. Wang S. Dong C. Wu Q. (2020). Exercise training modulates glutamic acid decarboxylase-65/67 expression through TrkB signaling to ameliorate neuropathic pain in rats with spinal cord injury. *Mol. Pain* 16:1744806920924511. 10.1177/1744806920924511 32418502 PMC7235678

[B50] Liem K. F. Tremml G. Jessell T. M. (1997). A role for the roof plate and its resident TGFbeta-related proteins in neuronal patterning in the dorsal spinal cord. *Cell* 91 127–138. 10.1016/s0092-8674(01)80015-5 9335341

[B51] Martinez M. Rossignol S. (2013). A dual spinal cord lesion paradigm to study spinal locomotor plasticity in the cat. *Ann. N. Y. Acad. Sci.* 1279 127–134. 23531010 10.1111/j.1749-6632.2012.06823.x

[B52] Mathis A. Mamidanna P. Cury K. M. Abe T. Murthy V. N. Mathis M. W. (2018). DeepLabCut: markerless pose estimation of user-defined body parts with deep learning. *Nat. Neurosci.* 21 1281–1289. 10.1038/s41593-018-0209-y 30127430

[B53] May Z. Fenrich K. K. Dahlby J. Batty N. J. Torres-Espín A. Fouad K. (2017). Following spinal cord injury transected reticulospinal tract axons develop new collateral inputs to spinal interneurons in parallel with locomotor recovery. *Neural Plast* 2017:1932875. 10.1155/2017/1932875 29138697 PMC5613456

[B54] Meisner J. G. Marsh A. D. Marsh D. R. (2010). Loss of GABAergic interneurons in laminae I-III of the spinal cord dorsal horn contributes to reduced GABAergic tone and neuropathic pain after spinal cord injury. *J. Neurotrauma* 27 729–737. 10.1089/neu.2009.1166 20059302

[B55] Mende M. Fletcher E. V. Belluardo J. L. Pierce J. P. Bommareddy P. K. Weinrich J. A. (2016). Sensory-derived glutamate regulates presynaptic inhibitory terminals in mouse spinal cord. *Neuron* 90 1189–1202. 10.1016/j.neuron.2016.05.008 27263971 PMC4912012

[B56] Nakajima H. Uchida K. Yayama T. Kobayashi S. Guerrero A. R. Furukawa S. (2010). Targeted retrograde gene delivery of brain-derived neurotrophic factor suppresses apoptosis of neurons and oligodendroglia after spinal cord injury in rats. *Spine* 35 497–504. 10.1097/BRS.0b013e3181b8e89b 20190624

[B57] Persson S. Boulland J. L. Aspling M. Larsson M. Fremeau R. T. Edwards R. H. (2006). Distribution of vesicular glutamate transporters 1 and 2 in the rat spinal cord, with a note on the spinocervical tract. *J. Comp. Neurol.* 497 683–701. 10.1002/cne.20987 16786558

[B58] Pfaff S. L. Mendelsohn M. Stewart C. L. Edlund T. Jessell T. M. (1996). Requirement for LIM homeobox gene Isl1 in motor neuron generation reveals a motor neuron-dependent step in interneuron differentiation. *Cell* 84 309–320. 10.1016/s0092-8674(00)80985-x 8565076

[B59] Rossignol S. (2006). Plasticity of connections underlying locomotor recovery after central and/or peripheral lesions in the adult mammals. *Philos. Trans. R. Soc. B Biol. Sci.* 361 1647–1671. 10.1098/rstb.2006.1889 PMC1664667 16939980

[B60] Rossignol S. Frigon A. (2011). Recovery of locomotion after spinal cord injury: some facts and mechanisms. *Annu. Rev. Neurosci.* 34 413–440. 10.1146/annurev-neuro-061010-113746 21469957

[B61] Roy R. R. Harkema S. J. Edgerton V. R. (2012). Basic concepts of activity-based interventions for improved recovery of motor function after spinal cord injury. *Arch. Phys. Med. Rehabil.* 93 1487–1497. 10.1016/j.apmr.2012.04.034 22920448

[B62] Rudomin P. (1999). Presynaptic selection of afferent inflow in the spinal cord. *J. Physiol. Paris* 93 329–347. 10574122 10.1016/s0928-4257(00)80061-3

[B63] Shah P. K. Garcia-Alias G. Choe J. Gad P. Gerasimenko Y. Tillakaratne N. (2013). Use of quadrupedal step training to re-engage spinal interneuronal networks and improve locomotor function after spinal cord injury. *Brain* 136 3362–3377. 10.1093/brain/awt265 24103912 PMC3808689

[B64] Sławińska U. Majczyński H. Dai Y. Jordan L. M. (2012). The upright posture improves plantar stepping and alters responses to serotonergic drugs in spinal rats. *J. Physiol.* 590 1721–1736. 10.1113/jphysiol.2011.224931 22351637 PMC3413485

[B65] Swieck K. Conta-Steencken A. Middleton F. A. Siebert J. R. Osterhout D. J. Stelzner D. J. (2019). Effect of lesion proximity on the regenerative response of long descending propriospinal neurons after spinal transection injury. *BMC Neurosci.* 20:10. 10.1186/s12868-019-0491-y 30885135 PMC6421714

[B66] Taccola G. Sayenko D. Gad P. Gerasimenko Y. Edgerton V. R. (2018). And yet it moves: recovery of volitional control after spinal cord injury. *Prog. Neurobiol.* 160 64–81. 10.1016/j.pneurobio.2017.10.004 29102670 PMC5773077

[B67] Takeoka A. Arber S. (2019). Functional local proprioceptive feedback circuits initiate and maintain locomotor recovery after spinal cord injury. *Cell Rep.* 27 71.e3–85.e3. 10.1016/j.celrep.2019.03.010 30943416

[B68] Takeoka A. Vollenweider I. Courtine G. Arber S. (2014). Muscle spindle feedback directs locomotor recovery and circuit reorganization after spinal cord injury. *Cell* 159 1626–1639. 10.1016/j.cell.2014.11.019 25525880

[B69] Tillakaratne N. J. K. de Leon R. D. Hoang T. X. Roy R. R. Edgerton V. R. Tobin A. J. (2002). Use-dependent modulation of inhibitory capacity in the feline lumbar spinal cord. *J. Neurosci.* 22 3130–3143. 10.1523/JNEUROSCI.22-08-03130.2002 11943816 PMC6757541

[B70] Tillakaratne N. J. K. Guu J. J. de Leon R. D. Bigbee A. J. London N. J. Zhong H. (2010). Functional recovery of stepping in rats after a complete neonatal spinal cord transection is not due to regrowth across the lesion site. *Neuroscience* 166 23–33. 20006680 10.1016/j.neuroscience.2009.12.010 PMC2820384

[B71] Tillakaratne N. J. K. Mouria M. Ziv N. B. Roy R. R. Edgerton V. R. Tobin A. J. (2000). Increased expression of glutamate decarboxylase (GAD67) in feline lumbar spinal cord after complete thoracic spinal cord transection. *J. Neurosci. Res.* 60 219–230. 10.1002/(SICI)1097-4547(20000415)60:2<219::AID-JNR11>3.0.CO;2-F 10740227

[B72] Torres-Espín A. Beaudry E. Fenrich K. Fouad K. (2018). Rehabilitative training in animal models of spinal cord injury. *J. Neurotrauma* 35 1970–1985. 30074874 10.1089/neu.2018.5906

[B73] Ying Z. Roy R. R. Edgerton V. R. Gómez-Pinilla F. (2005). Exercise restores levels of neurotrophins and synaptic plasticity following spinal cord injury. *Exp. Neurol.* 193 411–419. 15869943 10.1016/j.expneurol.2005.01.015

[B74] Zhang H. Liu Y. Zhou K. Wei W. Liu Y. (2021). Restoring Sensorimotor function through neuromodulation after spinal cord injury: progress and remaining challenges. *Front. Neurosci.* 15:749465. 10.3389/fnins.2021.749465 34720867 PMC8551759

[B75] Zimmerman A. L. Kovatsis E. M. Poszgai R. Y. Tasnim A. Zhang Q. Ginty D. D. (2019). Distinct modes of presynaptic inhibition of cutaneous afferents and their functions in behavior HHS public access. *Neuron* 102 420–434. 10.1016/j.neuron.2019.02.002 30826183 PMC6472967

