# Supplementary figures and images for: Changes in synaptic inputs to dI3 INs and MNs after complete transection in adult mice
Source: Front Neural Circuits. 2023 Jul 5;17:1176310. doi: 10.3389/fncir.2023.1176310 (PMC10354275; doi:10.3389/fncir.2023.1176310)

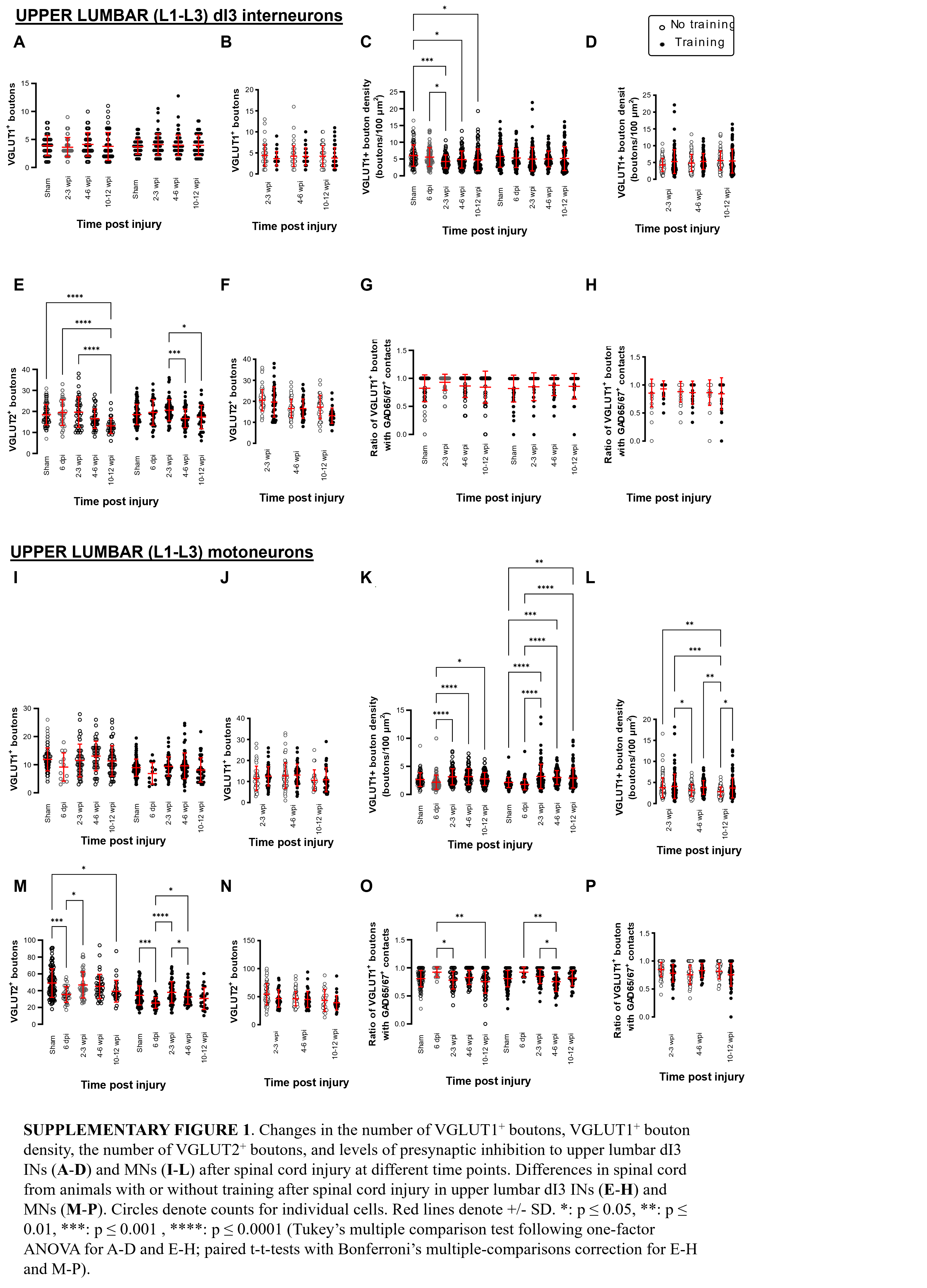

Supplement: Supplementary file 1 [file Image_1.tif]

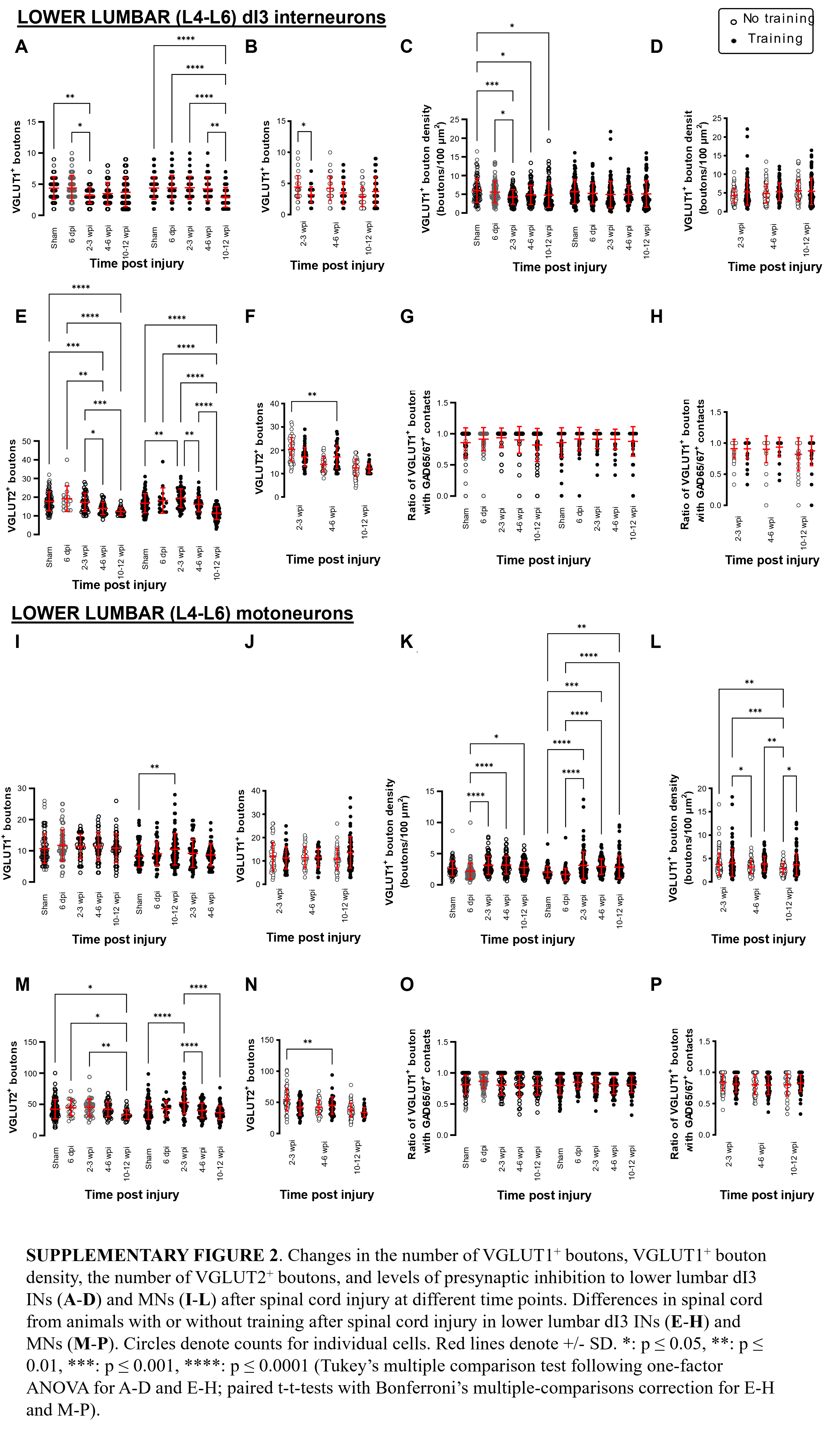

Supplement: Supplementary file 2 [file Image_2.tif]

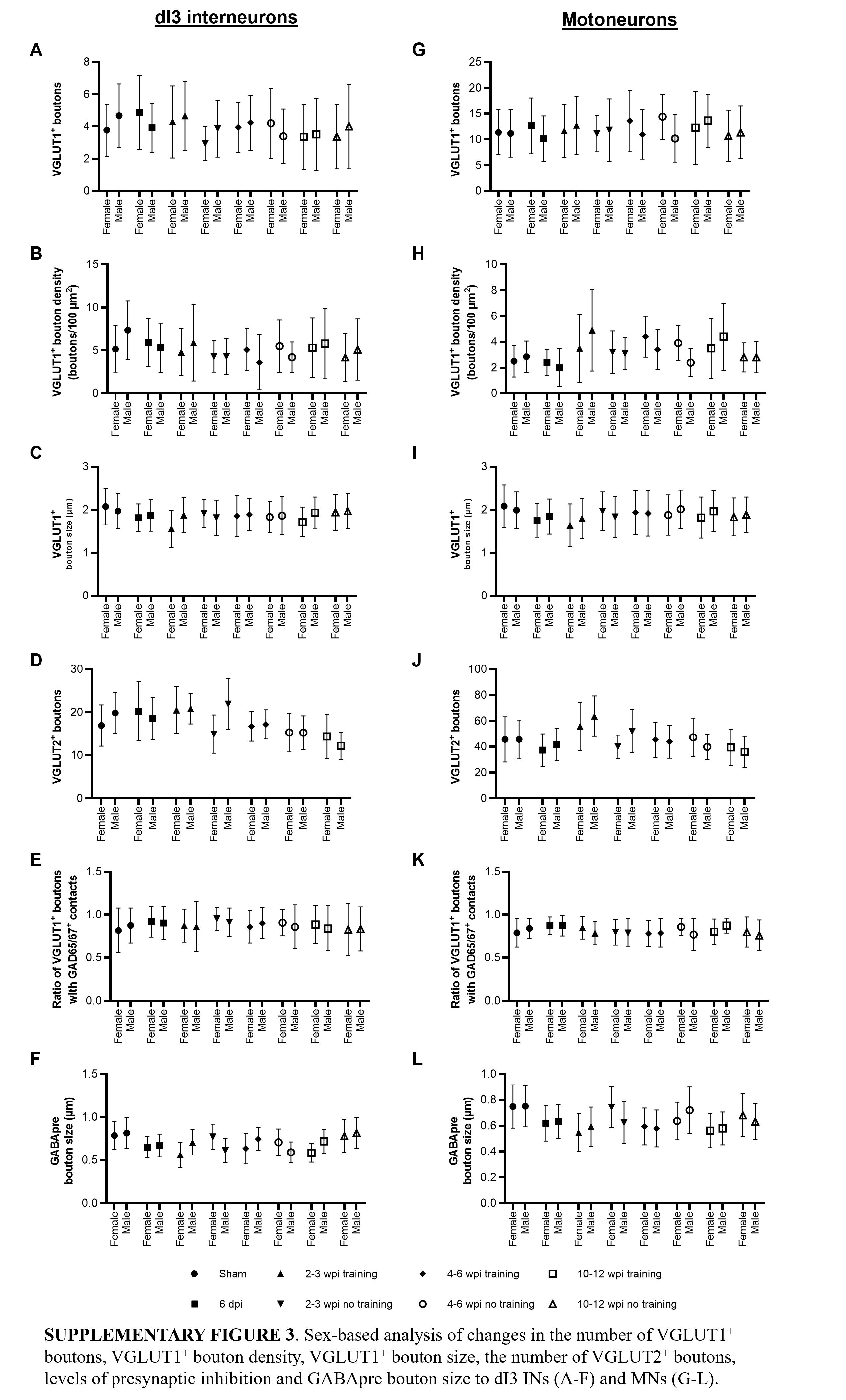

Supplement: Supplementary file 3 [file Image_3.TIF]
